# Scaling-up an mHealth system to deliver financial incentives to improve adherence to antiretroviral therapy in Tanzania

**DOI:** 10.1186/s43058-025-00766-1

**Published:** 2025-10-03

**Authors:** Emmanuel Katabaro, Babuu Joseph, Natalino Mwenda, Puspa Bhattarai, Janeth Msasa, Agatha Mnyippembe, Hamza Maila, Kassim Hassan, Jacqueline Kunesh, Amon Sabasaba, Solis Winters, Prosper Njau, Rebecca Hémono, Sandra I. McCoy, Laura Packel

**Affiliations:** 1 Health for a Prosperous Nation - a Non-Governmental, Nonprofit Research Organization Registered in Tanzania, Dar es Salaam, Tanzania; 2 Rasello - a Global Technology Company Specialized in Driving Digital Transformation Across Diverse Sectors, Including Healthcare, Governance, telecommunications, and education, Dar es Salaam, Tanzania; 3 School of Public Health, University of California, Berkeley, Berkeley, USA; 4 San Francisco, School of Nursing, University of California, San Francisco, USA; 5 University of Arizona College of Medicine - Phoenix, Phoenix, USA; 6 Tanzania Ministry of Health, Dodoma, Tanzania

**Keywords:** mHealth, Appointment reminders, People Living with HIV (PLHIV), 95–95-95 goals, Financial incentive, Implementation Science

## Abstract

**Background:**

Financial incentives are increasingly used to achieve UNAIDS' 95–95-95 goals for ending HIV by 2030. While evidence supports their effectiveness, scaling these interventions remains challenging. This study examines the implementation successes and challenges of a financial incentive intervention in Tanzania, delivered via an mHealth application that provides automated mobile money disbursements, biometric identification, and SMS reminders.

**Methods:**

Conducted alongside a Hybrid Type 1 Effectiveness-Implementation trial, the study evaluated financial incentives given to adults starting ART at 32 clinics. We used the Structured Assessment of Feasibility, Compatibility Beliefs in Technology (CBIT) scales, and the Program Sustainability Assessment Tool. Perspectives from 657 participants living with HIV and 90 clinic staff were collected using Proctor’s implementation science framework.

**Results:**

Clinic staff rated the mHealth system highly on CBIT subscales for perceived usefulness, ease of use, and compatibility, each scoring over 6 out of 7. Integration and applicability of the financial incentive within the mHealth system were well received, with 93.0% of staff agreeing it improved job performance. Among participants, 86.4% found SMS reminders helpful for attending appointments, and 76.7% felt the cash delivery met their expectations. Challenges included unreliable fingerprint identification and undelivered SMS reminders.

**Conclusions:**

Despite issues with fingerprint identification and SMS delivery, the financial incentive intervention via mHealth was found to be acceptable, feasible, and potentially sustainable in resource-limited settings, with support from host governments. Future research should enhance the intervention's effectiveness and optimize biometric identification methods.

**Trial registration:**

ClinicalTrials.gov NCT04201353. *Registered 17 December 2019, *https://clinicaltrials.gov/study/NCT04201353

**Supplementary Information:**

The online version contains supplementary material available at 10.1186/s43058-025-00766-1.

Contributions to the literature

- This is one of the largest trials of small, short-term financial incentives among Antiretroviral Therapy (ART) initiates to date, using a hybrid Effectiveness-Implementation design that allows for an evaluation of an implementation model at scale.
- The study evaluated a carefully designed mHealth system with several important functions: data collection; automatic cash incentive disbursements via mobile banking; biometric identification; SMS appointment reminders for patients; HIV blood sample collection and appointment reminders for clinic staff; and electronic, searchable medical and pharmacy records.
- Our results showed that delivering financial incentives through an mHealth system is an acceptable, feasible, and potentially sustainable approach for motivating adherence to ART in people living with HIV.

## Background

Financial incentives have shown significant promise in improving outcomes along the HIV care continuum for people living with HIV (PLHIV). A growing number of studies have demonstrated that offering financial incentives can improve retention in care [1–6], adherence to antiretroviral therapy (ART) [7, 8], and viral suppression [2, 5], and that they are a feasible and acceptable strategy for motivating engagement in HIV treatment [9]. Thus, financial incentives are increasingly recognized as a strategy that could help achieve the UNAIDS’ 95–95-95 goals [10], especially if brought to scale.

Digital and mobile health (mHealth) technologies have been increasingly adopted as an approach to improve HIV care delivery and patient outcomes [11, 12]. For example, appointment reminders delivered through SMS messages can improve ART adherence [13–15]; biometric patient identification also demonstrates potential to increase efficiency and decrease data errors [16–18]. In addition, previous studies reveal that clinicians and patients perceive mHealth systems to positively contribute to HIV care; however, some note concerns related to privacy and data sharing [19–22].

Relatively few studies have evaluated how financial incentives delivered via mHealth platforms can be sustainably implemented and scaled across broader health systems, particularly in low- and middle-income countries (LMICs). Recent reviews [23] have examined the scalability of mHealth HIV interventions and the application of financial incentives and gamification to improve adherence to medication for chronic conditions; however, there is limited evidence specifically focused on the delivery of financial incentives using mHealth for ART adherence.

We conducted a pilot study examining the effectiveness of financial incentives at four HIV clinics in the Lake Zone of Tanzania [2] delivered through an integrated mHealth system as the implementation strategy that included biometrics linked to patient electronic medical records, sending short messages for appointment reminders, and financial incentives delivered via mobile money transfer for attending HIV clinic visits. The results from this individually randomized trial demonstrated that the intervention and integrated mHealth system increased appointment attendance, retention in care, and viral suppression [2]. Interviews with clinicians and patients also revealed high adoption of the mHealth system alongside favorable perspectives of the platform. Questions also emerged about the scalability of the platform, particularly due to mobile device accessibility [24].

We subsequently investigated the effects of financial incentives on HIV care outcomes delivered using an optimized mHealth system at a larger scale, 32 HIV clinics in the Lake Zone [25]. The mHealth system used in this Hybrid Effectiveness-Implementation cluster randomized trial was refined based on patient and clinician feedback and included strategies to maximize clinic-level fidelity and adherence among PLHIV, such as SMS appointment reminders and HIV viral load sample collection notifications. Now, we aim to understand whether the elements of the mHealth system – automated mobile money distribution of financial incentives, biometric identification of PLHIV, SMS appointment reminders, clinic notifications – are feasible and acceptable to a broader sample of patients and clinicians and whether this implementation model could be scaled-up to facilitate integration of financial incentives for HIV treatment retention.

## Methods

### Study design

This implementation science study was conducted as part of a Hybrid Type 1 Effectiveness-Implementation trial of financial incentives for adults initiating antiretroviral therapy (ART) at 32 HIV primary care clinics, (“parent trial,” clinicaltrials.gov: NCT04201353) [26]. The parent trial was conducted from May 2021 to July 2023 and enrolled 1,990 ART initiates. The study sites included regional hospitals, district hospitals, health centers, and dispensaries located in urban and rural settings across the four regions (Geita, Mwanza, Kagera, and Shinyanga) in the Lake Zone of Tanzania, where HIV prevalence estimates range from 4.7% to 5.7% [27].

Eligible clinics were a minimum of 15 km from another study clinic, had at Least 65 new ART initiates per quarter, were within 100 km driving distance of Bukoba, Mwanza, Geita, Kahama, or Shinyanga cities, and used an electronic medical records database. Study sites were randomized 1:1 to the intervention arm, which provided up to 6 months of financial incentives conditional on completing a monthly clinic visit, or to the comparison arm, which provided standard HIV services as outlined by the Ministry of Health. The aim of the parent trial was to evaluate the effectiveness of the financial incentive intervention on ART retention and viral suppression. The primary outcome was retention on ART with viral suppression (< 1,000 cp/ml) at 12 months. The effectiveness portion of the study found that the intervention modestly improved the proportion of PLHIV on ART and with viral suppression at 6 and 12 months [25].

### Financial incentive intervention

Monthly financial incentives of 22,500 TSH (~ $10 USD) were provided to PLHIV in the intervention arm via the mHealth system and integrated into clinic workflow. The incentives were disbursed automatically to patients’ registered mobile money phone numbers. The intervention was designed to follow the country’s model of HIV care and treatment service delivery, whereby all new ART initiates are dispensed ART during monthly clinic visits for the first six months until they have a suppressed HIV viral load test result. Since the first six months are often the most difficult for new initiates, leading to poor adherence and disengagement from care [28], the intervention was designed to provide much-needed support to these patients during this critical time.

### mHealth system

Both study arms implemented an mHealth system for data collection (all clinics) and incentive disbursement (intervention clinics only), that was refined based on findings from our previous study [24] to include patient SMS appointment reminders and HIV viral load sample collection notifications to clinic staff. This included improvements to ensure the mHealth system fits the needs of the study and clinic environment. The system included the following elements:

1. Fingerprint scanning for biometric identification of PLHIV (all sites). The fingerprint scanner was one of two possible ways to register a person’s visit attendance and was intended to save time and improve accuracy over manually entering a 14-digit clinic ID.
2. Automatic mobile money disbursement of incentives (intervention sites) for eligible clinic visits.
3. SMS appointment reminders 7 days and 1 day prior to scheduled appointments (all sites).
4. Reminders for clinic staff to conduct viral load testing at required time points (all sites); and
5. Appointment attendance tracking (all sites).

The mHealth system, loaded on tablet computers, was operated by trained clinic staff with periodic supervision from research staff.

### Implementation science study participants

The implementation science study included a subset of participants (*n* = 657 of 1,990 PLHIV) from the parent trial who met the following criteria: ≥ 18 years, initiated ART in the prior 30 days (during parent trial enrollment), not planning to relocate within 12 months of study enrollment, and willing to provide informed consent. Surveys were conducted as part of routine procedures with trial participants at their 6-month study visit, which included questions about aspects of the mHealth system and the financial incentive intervention (intervention arm only).

The implementation study also included a sample of clinic staff (*n* = 90) from the 32 HIV clinics who were key in the active enrollment and follow-up of patients in the parent trial by operating the mHealth system, completing study procedures, and providing standard HIV care and treatment services. Eligible clinic staff were: working at any Level and department of one of the 32 HIV Care and Treatment Center (CTC) sites, knowledgeable about study procedures and/or trained in using the mHealth system, and willing to provide informed consent. Clinic staff, including the CTC in-charge, pharmacists, general physicians, nurses, laboratory personnel, and general staff, including home based care workers, were purposely sampled to represent the perspectives of all levels of clinic organization.

### Implementation outcomes

The study outcomes were guided by Proctor’s Implementation Science Framework [29] and included fidelity, acceptability/appropriateness, feasibility, and sustainability (Table 1). And used the following validated scales to assess these outcomes among clinic staff: Compatibility Beliefs in Technology Acceptance (CBIT) scale [30] (appropriateness and compatibility of the mHealth system); the Structured Assessment of Feasibility scale [31] (SAFE; feasibility of implementation of both the mHealth system and the financial incentive intervention); and the Program Sustainability Assessment Tool [32] (PSAT; sustainability of the intervention in facility settings). In addition, some questions were included in both the patient and clinic staff surveys that were specific to the study and not part of a validated scale. Backend data from the mHealth system was used to assess intervention fidelity related to timely delivery of financial incentives, timely sending and delivery of SMS messages, and successful utilization of fingerprint scanner.

**Table 1:** Implementation science outcomes, definitions and data sources

IS Outcome	Measure	Level	Data Source
Fidelity	Proportion of visits for which patients reported to have registered in the mHealth during clinic visits	Patient	6-month survey with patients
	Proportion of visits for which the mHealth system was used as intended in registration and pharmacy	Patient	
	Proportion of visits for which the fingerprint scan recognition was successfully used (e.g., no manual entry of patient ID needed, or only one attempt needed)	Patient	Backend system data
	Proportion of SMS messages that were sent successfully (delivered and sent at the correct time(s)	Patient	Backend system data
Acceptability /Appropriateness	*Compatibility Beliefs in Technology Acceptance* scale	Clinic	12-month survey with clinic staff
	Perspectives on the fingerprint scanner	Clinic	
	Perspectives on the sustainability of financial incentive intervention	Clinic	
Acceptability /Appropriateness	Patient perspectives on clinic interaction	Patient	6-month survey with patients
Feasibility	*Structured Assessment of Feasibility* scale	Clinic	12-month survey with clinic staff
Sustainability	*Program Sustainability Assessment Tool*	Clinic	12-month survey with clinical staff

### Analysis

Data were analyzed in R Studio (version 4.3.0) [33]. Descriptive statistics were compared and stratified by sex and study arm, including scores for the CBIT [30] and PSAT scales [32].

The CBIT scale includes six subscales evaluating perceived usefulness, ease of use, compatibility with values, compatibility with preferred work style, compatibility with existing practices, and compatibility with prior experience. Each subscale was scored individually, and scores ranged from 0—7; a higher score corresponds to a more favorable belief related to acceptability and appropriateness of the mHealth system.

The PSAT measure was adapted for our study to include four of the eight sustainability domains for both the mHealth system and financial incentive intervention: environmental support (internal and external climate supportive of the program), funding sustainability (consistent, reliable funding for the program), partnerships (stakeholder support), and organizational capacity (internal support and resources to manage program). The PSAT uses a 1–7 Likert scale, with 1 indicating 'to little or no extent' and 7 indicating 'to a great extent'; a higher score indicates more potential for sustainability. For the purposes of this study, we modified the scale to include 0 to represent 'true to no extent,' providing respondents with an option to indicate if they did not perceive the statement to be true at all, and 1 to represent ‘true to a little extent’. An average score was calculated for each domain, with weighting used to account for missingness. The SAFE scale includes statements assessing barriers and facilitators to program implementation. The scale items were evaluated individually rather than scored overall, per existing recommendations [31]. Means and standard deviations (SD) were calculated for non-scale items included in the patient and clinic staff surveys assessing fidelity and appropriateness (especially as it related to biometric identification), as well as for the mHealth backend data.

### Ethics

The study was funded by the U.S. National Institute of Health (NIH) and ethical approvals were obtained from the National Institute for Medical Research (NIMR), Tanzania and the Committee for Protection of Human Subjects (CPHS) at the University of California, Berkeley. Study staff were trained in ethical guidelines for human subjects research.

## Results

### Demographic characteristics of the study population

The implementation science study included 657 PLHIV participating in the parent trial and 90 clinic staff. Patients were 35 years on average, 62.0% female, 48.2% had completed primary school, 60.0% were married or partnered, and 60.0% had worked in the past 7 days (Table 2). Characteristics of the subset of PLHIV in the implementation science study were similar to the overall sample included in the parent trial [25]. The clinic staff comprised registration staff who are the first contact with people at the clinic and who were the primary staff members using the mHealth system (32.2%), CTC in-charge staff (26.7%), and pharmacists who played a key role in dispensing ART, authorizing delivery of financial incentives, and entering data necessary for appointment reminders in the mHealth system (26.7%).

**Table 2:** Baseline characteristics of study participants and clinic staff in the implementation science study, overall and by study arm, Tanzania 2021–2022

**Participants living with HIV**	**Overall** **(*N*** **= 657)**	**Comparison** **(*N*** **= 385)**	**Intervention** **(*N*** **= 272)**
***Demographics***			
Age, years	35.98 (11.48)	35.99 (11.26)	35.96 (11.81)
Female (%)	407 (61.9)	233 (60.5)	174 (64.0)
Married or partnered (%)	389 (60.0)	236 (62.4)	153 (56.7)
Head of household (%)	367 (56.6)	231 (61.1)	136 (50.4)
Language (%)			
*Swahili*	222 (34.3)	127 (33.6)	95 (35.2)
*Sukuma*	289 (44.6)	175 (46.3)	114 (42.2)
*Haya*	117 (18.1)	70 (18.5)	47 (17.4)
*Other*	20 (3.1)	6 (1.6)	14 (5.2)
***Socioeconomic status***			
Educational attainment (%)			
* No formal education*	163 (25.2)	95 (25.1)	68 (25.3)
* Some primary*	97 (15.0)	55 (14.6)	42 (15.6)
* Completed primary*	312 (48.2)	182 (48.1)	130 (48.3)
* More than primary*	75 (11.6)	46 (12.2)	29 (10.8)
Worked in the past 7 days (%)	398 (61.4)	235 (62.2)	163 (60.4)
Household size	4.55 (3.00)	4.52 (3.17)	4.58 (2.76)
Moderate or severe household hunger (HHS-3) (%)	40 (6.1)	24 (6.3)	16 (5.9)
Wealth score	6.37 (7.59)	6.63 (8.26)	5.99 (6.52)
***Health & HIV Care***			
Depression (PHQ-2) (%)	411 (63.4)	249 (65.9)	162 (60.0)
Anxiety (GAD-2) (%)	380 (58.6)	224 (59.3)	156 (57.8)
Days on ART at enrollment	3.38 (6.54)	3.42 (6.80)	3.33 (6.17)
DTG-based regimen (%)	654 (100.0)	383 (100.0)	271 (100.0)
Weight (kg)	57.12 (9.59)	57.12 (8.99)	57.13 (10.41)
WHO Clinical Stage^a^ (%)			
Stage 1	482 (73.4)	300 (77.9)	182 (66.9)
Stage 2	123 (18.7)	60 (15.6)	63 (23.2)
Stage 3	41 (6.2)	19 (4.9)	22 (8.1)
Stage 4	4 (0.6)	2 (0.5)	2 (0.7)
Missing	7 (1.1)	4 (1.0)	3 (1.1)
Pregnant (%)	29 (7.1)	20 (8.3)	9 (5.3)
**Clinic staff**	**Overall** **(*N* = 90)**	**Comparison** **(*N* = 48)**	**Intervention** **(*N* = 42)**
***Role***			
Registration staff	29 (32.2)	14 (29.2)	15 (35.7)
CTC in-charge	24 (26.7)	14 (29.2)	10 (23.8)
Pharmacist	24 (26.7)	14 (29.2)	10 (23.8)
Nurse or Physician	8 (8.9)	3 (6.2)	5 (11.9)
Pharmacy staff	2 (2.2)	2 (2.2)	0 (0.0)
Other	3 (3.3)	1 (2.1)	2 (4.8)

Data are n(%) or mean(SD). *ART* Antiretroviral therapy, *HHS* Household Hunger Scale, *PHQ* Patient Health Questionnaire, *GAD* Generalized Anxiety Disorder, *WHO* World Health Organization, *DTG* Dolutegravir, *CTC* Care and Treatment Center

^a^The WHO clinical staging system is a standardized approach to classifying HIV-related disease progression in adults and adolescents, especially useful where access to laboratory diagnostics is limited. It categorizes the clinical conditions associated with HIV into four stages to guide clinical management and treatment decisions

### Fidelity

#### mHealth system, PLHIV perspectives

Patients reported consistent use of the tablet-based mHealth system by clinic staff for visit check-in. Overall, 461 (70.2%) patients reported that they used a tablet computer with the mHealth system to check-in for every clinic visit. Only 19 (2.9%) never checked in for their visit on a tablet (Supplemental Table 1). There were no notable differences in the proportion of patients who checked in using the mHealth system by study arm.

Most patients surveyed (*n* = 398, 60.6%) reported using the fingerprint scanner (rather than manually entering a 14-digit ID) to check in at every visit, with no notable differences between the two study arms. Two (0.3%) participants reported that they had never scanned their fingerprints at the clinic. There was substantial variation by study region in the proportion of visits where the fingerprint scanner was used (median: 65.3%), range (49.4%—86.5%). In addition, 73.5% of patients disagreed or strongly disagreed with the statement that the fingerprint scanner was unreliable, with no notable differences by sex or study arm.

#### mHealth system, clinic staff perspectives

There was mixed feedback from clinic staff with respect to the use of fingerprint scanners. According to a clinic staff survey, use was inconsistent across clinics, with an equal number of clinic staff agreeing 34 (39.5%) and disagreeing 34 (39.5%) with the statement that they rarely used the fingerprint scanner. There were also mixed perspectives on the reliability of the fingerprint scanner; 37 (43.0%) clinic staff reported that the scanner did not work as expected, while 29 (33.7%) disagreed with this statement. The fingerprint scanning was more beneficial for some clinic staff than others, with 40 (46.6%) clinic staff agreeing that the fingerprint scanner helped check-in study participants more quickly, and 24 (27.9%) clinic staff disagreeing with this statement. Approximately half of clinic staff (*n* = 55, 56.0%) agreed that using the fingerprint scanner provided too many possible matches, most of which did not include the targeted scanning participant, and that it was easier to enter the participants’ ID number rather than use the fingerprint scanner (*n* = 51, 59.4%). Despite these challenges, 67 (77.9%) clinic staff agreed or strongly agreed that it was easy to get the mHealth system to do what they wanted, including the flexibility to manually enter a participant’s ID in cases of fingerprint scan failures.

#### mHealth system, backend system data

The backend data from the mHealth system revealed that on average, only 11% of visits had a successful fingerprint scan on the first attempt (median: 11.2), (range: 8—17%). When using repeated attempts, 35% (median: 33.2) of visits had a successful fingerprint scan (range: 11—83%).

All patients in the parent trial were sent SMS visit reminder messages. Backend data from the mHealth system revealed that, on average, 95% of the expected SMS appointment reminders (two per visit) were sent from the system on time (one message sent 7 days before and one message sent 1 day before the visit) at each clinic site (range: 90–99% across 32 sites). However, only 54% of expected SMS messages were reported as “delivered” to mobile phones on time at each site (range: 41–67% across 32 sites), potentially due to issues with mobile network providers.

### Acceptability and appropriateness

#### mHealth system, clinic staff perspectives

In general, there were high levels of perceived acceptability/appropriateness of the mHealth system based on the CBIT scores; however, there was some variation by subscale (Table 3). Perceived usefulness, ease of use, compatibility with preferred work style, and compatibility with values all had average scores above 6 (with a maximum of 7) indicating high Levels of acceptability across these domains. For example, 85 (94.4%) respondents agreed or strongly agreed that: 1) “using the mHealth system enhanced my effectiveness in providing HIV care”; 2) using the mHealth system made it easier to do their job providing care for PLHIV; and 3) using the mHealth system had improved their job performance (perceived usefulness). 79 (87.8%) agreed or strongly agreed that “Using the mHealth system fits my preferred method for doing my job” (compatibility with preferred work style). The score on the compatibility with existing practices subscale was 4.8, where 51 (56.7%) of respondents agreed or strongly agreed that “To use the mHealth system, I don’t need to change anything I do”. Compatibility with prior experience showed the mHealth system was a new work experience for more than half of facility staff (*n* = 64 (71.1%), with a mean score of 3.6).

**Table 3:** Compatibility Beliefs in Technology assessment of the mHealth system (CBIT scale) among clinic staff in the Lake Zone, Tanzania 2021–2022

[Compatibility Beliefs in Technology Scale (CBIT), Subscales]	(*N* = 90)
**Perceived usefulness**	
Using the mHealth system in my job has increased my productivity	86 (95.6%)
Using the mHealth system has made it easier to do my job providing care for people living with HIV	86 (95.6%)
Using the mHealth system has improved my job performance	86 (95.6%)
Using the mHealth system has enhanced my effectiveness in providing HIV care	85 (94.4%)
**Score**	**6.38 (0.677)**
**Ease of use**	
My interaction with the mHealth system is clear and understandable	84 (93.3%)
I find the mHealth system to be flexible to interact with	83 (92.2%)
It has been easy for me to become skillful at using the mHealth system	81 (90.0%)
I find the mHealth system easy to use	80 (88.9%)
Learning to operate the mHealth system has been easy for me	74 (82.2%)
I find it easy to get the mHealth system to do what I want	71 (78.9%)
**Score**	**6.28 (0.707)**
**Compatibility with Preferred Work Style**	
Using the mHealth system fits my preferred method for doing my job	79 (87.8%)
Using the mHealth system fits my preferred routine	78 (86.7%)
Using the mHealth system fits well with the way I like to work	78 (86.7%)
The mHealth system enables me to work in the way I prefer	77 (85.6%)
**Score**	**6.13 (0.750)**
**Compatibility with Existing Practices**	
Using the mHealth system is compatible with most aspects of the way I typically conduct my job	72 (80.0%)
Using the mHealth system does not require significant changes in my existing work routine	65 (72.2%)
To use the mHealth system, I don't have to change anything I currently do	51 (56.7%)
Using the mHealth system has required a change in the way that I conduct my job	35 (38.9%)
Using the mHealth system has forced me to change my existing method of conducting my job	28 (31.1%)
**Score**	**4.81 (1.03)**
**Compatibility with prior experience**	
Using the mHealth system does not require significant changes in my existing work routine	65 (72.2%)
Using the mHealth system has been a new experience for me	64 (71.1%)
Using the mHealth system is compatible with my past computer experience	57 (63.3%)
Using the mHealth system is a new work experience for me	56 (62.2%)
Using the mHealth system is different from other experiences I have had	46 (51.1%)
Using the mHealth system is different from using other software I have used in the past	37 (41.1%)
Using the mHealth system is not similar to anything that I've done before	31 (34.4%)
**Score**	**3.60 (1.30)**
**Compatibility with values**	
Use of the mHealth system is consistent with the way I think business should be conducted	79 (87.8%)
Using the mHealth system is not appropriate for a person with my values regarding the role of technology	11 (12.2%)
Using the mHealth system does not fit the way I view the world	9 (10.0%)
Using the mHealth system goes against what I believe technology should be used for	7 (7.8%)
Using the mHealth system runs counter to my own values	4 (4.4%)
Using the mHealth system runs counter to my values about how to conduct my job	4 (4.4%)
**Score**	**6.16 (0.087)**

Data are presented as frequency and (%) who agree/strongly agree with each sub domain statement; Scores are presented as mean (SD)

#### Cash intervention, patient perspectives

On participants’ satisfaction with the delivery of cash incentives (intervention participants only), 171 (76.7%) of participants in the intervention arm agreed that the delivery of cash met their expectations, 17 (7.6%) disagreed, and 15.2% reported that they neither agreed nor disagreed with this statement. There was heterogeneity in satisfaction with the overall delivery of cash across the study regions as follows: Mwanza 62 (67.4%), Kagera 41 (76.0%), and Geita 67 (88.2%). On the perspective that the time to use the tablet was “worth it” to receive the incentive at the end of the visit, overall, 173 (77.6%) of 223 patients agreed while only 19 (8.5%) disagreed with this statement, and 31(13.9%) were neutral.

### Feasibility

#### SAFE scale—mHealth system, clinic staff perspective

The majority (91.1%) of clinic staff agreed that the mHealth system is flexible and can be tailored to the context and situation of the daily clinic activities; 90.0% agreed that it made their work significantly faster and more efficient, and 68.9% agreed that using the mHealth system required Less than 30 min of additional work per week (Table 4). Most (88.9%) clinic staff agreed that the mHealth system had no known associated adverse events, while just over half (51%) agreed that the mHealth system could save costs. However, very few staff agreed that using the system required no training (4.4%) or additional resources (18.9%), or that the system cost was low (37.8%). About half of clinic staff (48%) agreed that there would be a need for additional staff to implement the mHealth system.

**Table 4:** Feasibility Assessment of the mHealth system and financial incentives (SAFE scale) among clinic staff in the Lake Zone, Tanzania 2021–2022

Structured Assessment of Feasibility (SAFE) scale *mHealth*	Agreement with Statement (n, %) (*N* = 90)
The mHealth system is flexible and can be tailored to the context and situation	82 (91.1%)
The mHealth system makes my work significantly faster and more efficient	81 (90.0%)
There are no known adverse events associated with the mHealth system	80 (88.9%)
The mHealth system requires Learning only 1 new process	64 (71.1%)
Using the mHealth system requires < 30 min per week of additional work	62 (68.9%)
Our clinic does not require additional staff to use the mHealth system	48 (53.3%)
The mHealth system has been demonstrated to save costs	46 (51.1%)
The mHealth system cost is low	34 (37.8%)
The mHealth system does not require any additional support visits/sessions	29 (32.2%)
The mHealth system implementation does not require any additional resources	17 (18.9%)
Using the mHealth system does not require any specific training	4 (4.4%)
**Financial Incentive**	**(*N* = 42)**
The financial incentive intervention has been designed for the population of interest	41 (97.6%)
The financial incentive intervention is flexible and can be tailored to the context and situation	35 (83.3%)
The financial incentive intervention will very likely improve retention and viral suppression	35 (83.3%)
Implementing the financial incentive intervention requires < 30 min per week of additional work	33 (78.6%)
Our clinic does not require additional staff to implement the financial incentive intervention	31 (73.8%)
There are no know adverse events associated with the mHealth system	30 (71.4%)
The financial incentive intervention requires Learning only 1 new process	29 (69.0%)
The financial incentive intervention has been demonstrated to save costs	29 (69.0%)
Implementing the financial incentive intervention does not require any additional resources	19 (45.2%)
The cost of the financial incentive intervention is low	18 (42.9%)
Implementing the financial incentive intervention does not require any additional support visits/sessions	13 (31.0%)
Delivering the financial incentive intervention does not require any specific training	12 (28.6%)

Data are presented as frequency and (%) who agree with each sub domain statement

#### SAFE scale—financial incentive intervention, clinic staff perspectives

Clinic staff at intervention clinics were asked about their perspectives on different aspects of the financial incentive intervention. The majority (97.6%) of clinic staff reported that the financial incentive intervention was designed for the population of interest. Most (83.3%) agreed that the financial incentive intervention is flexible and can be tailored to the situation and context in the clinic, and that it is likely to improve retention and viral suppression. Nearly 70% of clinic staff were positive about whether the financial incentive intervention could eventually save costs over time, but 38.1% agreed that the financial incentive intervention was likely to be too costly to provide without additional funding. Statements on the financial incentive intervention requiring additional material resources (e.g., equipment) or ongoing supervision and support were supported by 19 (45.2%) and 13 (31.0%) clinic staff, respectively.

### Sustainability

#### Program Sustainability Assessment Tool (PSAT)—mHealth system, clinic staff perspectives

Overall, the four PSAT domains (Table 5) for mHealth demonstrate moderately high capacity for sustaining the mHealth system. The partnerships domain had the highest mean score (5.7) indicating that the clinics and clinic authorities have developed partnerships with government partners and other stakeholders in effort to sustain the mHealth system. Environmental support (5.6) and organizational capacity (5.6) received similar scores, while funding sustainability (5.0) scored lower.

**Table 5:** Program Sustainability Assessment (PSAT) of the mHealth system and financial incentives among Clinic Staff in the Lake Zone, Tanzania 2021–2022

Program Sustainability Assessment Tool *(mHealth system)*	Overall (*N* = 90)
**Environmental Support**	
Mean (SD)	5.6 (1.1)
Median [Min, Max]	6.0 [1.5, 7.0]
**Partnerships**	
Mean (SD)	5.7 (1.3)
Median [Min, Max]	6.0 [1.0, 7.0]
**Funding Sustainability**	
Mean (SD)	5.0 (1.4)
Median [Min, Max]	5.3 [1.0, 7.0]
**Organizational Capacity**	
Mean (SD)	5.6 (1.1)
Median [Min, Max]	6.0 [2.6, 7]
**(Financial Incentive)**	**Overall (*N* = 42)**
**Environmental Support**	
Mean (SD)	5.9 (1.7)
Median [Min, Max]	5.38 [1.5, 7.0]
**Partnerships**	
Mean (SD)	4.2 (2.2)
Median [Min, Max]	4.5 [0.0, 7.0]
**Funding Sustainability**	
Mean (SD)	4.5 (1.8)
Median [Min, Max]	4.8 [0.0, 7.0]
**Organizational Capacity**	
Mean (SD)	6.0 (1.1)
Median [Min, Max]	6.2 [2.4, 7.0]

The maximum score for each domain is 7

#### PSAT—financial incentives, clinic staff perspectives

The PSAT domains for financial incentives revealed a similar pattern, suggesting moderate capacity for sustaining the financial incentive intervention. The organizational capacity and environmental support domain scored the highest (6.0 and 5.9 on average, respectively), while the partnership and funding sustainability domains scored the lowest (4.2 and 4.5, respectively).

## Discussion

Although evidence about the effectiveness of financial incentives for the HIV care continuum is growing, there is limited information about scalable implementation models to implement incentive programs in clinic settings. To address this gap, we explored Proctor’s Implementation Science Outcomes in relation to two aspects of the intervention implementation strategy: the mHealth system and delivery of the financial incentive. Overall, our findings suggest a high level of fidelity to and use of the mHealth system by both clinic staff and PLHIV attending appointments. We also observed high levels of acceptance of the system. In contrast, the degree of fidelity with respect to use of the fingerprint scanner was relatively low; this observation could be attributed to some clinics stopping the use of fingerprint scans due to persistent technology challenges whereby recognition of fingerprints was impeded due to unstable internet connectivity, poor image quality, and suboptimal precision in identifying participants. However, the majority (73.5%) perceived fingerprint scanners as reliable, which reflects an important shift in user attitudes towards fingerprint scanners suggesting increased willingness to use them contrary to findings from previous studies [34].

We also found a high success rate of sending SMS appointment reminders among patients in both intervention and control groups; however, the SMS receiving rate was low. Previous studies have also demonstrated that SMS messages are feasible and acceptable to improve engagement in HIV care in Tanzania [9, 24, 26]. However, some hurdles still remain [35]. Nevertheless, incorporating SMS messages into routine HIV care has the potential to improve treatment engagement at scale given their low cost and acceptability and feasibility among PLHIV. This supports the use of low-cost digital interventions to strengthen care engagement and suggests that with improved delivery mechanisms, SMS-based systems can become an integral component of national HIV care strategies.

Further, the study revealed that there is potential for scaling the mHealth system and financial incentive intervention if the reported limitations are effectively addressed. The most notable challenge was unreliable biometric identification, which has also been observed in other studies in resource-constrained settings [36, 37]. Importantly, the basic infrastructure for implementing the financial incentive intervention is already in place as there is a rapid increase in the use of mobile money among people in Tanzania [38]. Biometric systems, particularly fingerprint scanners, have already been adopted and integrated into routine HIV service delivery in select regions of Tanzania, where they have become standard practice in many HIV clinics. This reflects a broader shift toward digital health technologies, and demonstrates their potential to improve patient identification and continuity of care. However, to fully realize this potential, it is essential to implement contingency plans for technological failures, such as providing staff training on troubleshooting, and ensuring regular maintenance and updates of devices. These measures can help mitigate disruptions in service delivery and support the sustainable integration of digital tools into health systems.

Taken together, these results suggest: i) clinic staff are welcoming of the system because it simplifies their day-to-day activities and maximizes efficient handling of data; ii) periodic technical support should be provided to ensure smooth implementation; iii) there is a definitive need to ensure PLHIV have access to mobile phones and mobile money; and iv) a strong government involvement for the sustainability of the financial incentive program as partnerships and funding sustainability may not be reliable. These results are reinforced by findings from previous studies which have shown that assessing and planning for sustainability is particularly critical in low-resource settings where various programs are implemented with time-limited funding from external donors [23].

The findings from our study have indicative promising implications for wider application in other low-and middle-income countries (LMICs), especially those with similar health system structures, digital infrastructure, and disease burden. Previous findings from LMICs are indicative of the implementation challenges similar to our study findings giving a snapshot of the similarity of the shared operating settings into which they can be generalized [39, 40]. The demonstrated acceptability and feasibility of using mHealth tools and financial incentives to support HIV care retention in Tanzania from a rigorous implementation study, suggests that similar models could be adopted in other LMICs where mobile phone ownership and mobile money use are increasing [41–43]. However, technical support, contingency plans for technology failure, and government ownership are likely applicable to other contexts and should be considered to ensure sustainability.

Furthermore, the integration of financial incentives within existing health systems using widely adopted and accepted tools like mobile money enhances the scalability and relevance of this approach across settings with similar resource limitations. However, successful replication elsewhere will require some adjustments depending on the specific context, stakeholder engagement beyond clinic staff, and planning for long-term sustainability beyond donor funding [44].

It is important to acknowledge some limitations of the study. Some scales used were not validated in the local settings, the study did not include stakeholders outside of clinic staff, and we were only able to survey a subset of patient participants for the implementation science related questions.

## Conclusion

Financial incentives delivered via a clinic-based mHealth system can provide a springboard for retaining PLHIV in care. Some financial incentive programs are underway in low-resource settings, Tanzania included, and have demonstrated positive impact. However, a feasible, acceptable, and scalable implementation model is essential to realize these benefits outside a research study setting. The model evaluated here has potential to be scaled as our results provide evidence that financial incentives for retention in HIV care provided through an mHealth system are an effective, feasible and acceptable approach.

In summary, while these findings are most directly applicable to settings like Tanzania, the main components of the intervention; mHealth systems, mobile financial incentives, and strategic support, have significant potential for generalization to other LMICs, provided implementation challenges are proactively addressed.

## Supplementary Information

Supplementary Material 1.

## Data Availability

The datasets used and analyzed during the current study are available from the corresponding author on reasonable request.

## Abbreviations

PSAT: Program Sustainability Assessment Tool;
ART: Antiretroviral therapy;
SAFE scale: Structured Assessment of Feasibility;
HIV: Human Immunodeficiency Virus;
CBIT: Compatibility Beliefs in Technology
