# Supplementary material for: Scaling-up an mHealth system to deliver financial incentives to improve adherence to antiretroviral therapy in Tanzania
Source: Implement Sci Commun. 2025 Oct 3;6:100. doi: 10.1186/s43058-025-00766-1 (PMC12495787; doi:10.1186/s43058-025-00766-1)
Supplement: Supplementary file 1 — Supplementary Material 1. [file 43058_2025_766_MOESM1_ESM.docx]

**Supplemental Table 1: PLHIV Patient Perspectives on use of the mHealth system at Appointment**

|  | **Control**  **(N=385)** | **Treatment**  **(N=272)** | **Overall**  **(N=657)** |
| --- | --- | --- | --- |
| **When you came to the clinic for your regular appointments since you enrolled in the study, how often were you checked in with the tablet computer?** | | | |
| At every clinic visit | 68.1% (262) | 73.2% (199) | 70.2% (461) |
| At some clinic visits | 27.3% (105) | 45 (16.5%) | 22.8% (150) |
| Never checked in with the tablet | 1.8% (7) | 4.4% (12) | 2.9% (19) |
| **When you came to the clinic for your regular appointments since you enrolled in the study, how often did you scan your fingerprint?** | | | |
| At every clinic visit | 60.0% (231) | 61.4% (167) | 60.6% (398) |
| At some clinic visits | 35.3% (136) | 27.6% (75) | 32.1% (211) |
| Never scanned my fingerprint | 0% (0) | 0.7% (2) | 0.3% (2) |
| Missing data: Checked in with tablet (n=27), scanned fingerprint (n=46) | | | |
